# Core-Shell Beads Made by Composite Liquid Marble Technology as A Versatile Microreactor for Polymerase Chain Reaction

**DOI:** 10.3390/mi11030242

**Published:** 2020-02-26

**Authors:** Kamalalayam Rajan Sreejith, Lena Gorgannezhad, Jing Jin, Chin Hong Ooi, Takayuki Takei, Gen Hayase, Helen Stratton, Krystina Lamb, Muhammad Shiddiky, Dzung Viet Dao, Nam-Trung Nguyen

**Affiliations:** 1 Queensland Micro- and Nanotechnology Centre, Nathan Campus, Griffith University, 170 Kessels Road, Brisbane, QLD 4111, Australia; sreejith.kamalalayamrajan@griffithuni.edu.au (K.R.S.); lena.gorgannezhad@griffithuni.edu.au (L.G.); jing.jin3@griffithuni.edu.au (J.J.); c.ooi@griffith.edu.au (C.H.O.); h.stratton@griffith.edu.au (H.S.); k.lamb@griffith.edu.au (K.L.); m.shiddiky@griffith.edu.au (M.S.); d.dao@griffith.edu.au (D.V.D.); 2 School of Environment and Science, Nathan Campus, Griffith University, 170 Kessels Road, Brisbane, QLD 4111, Australia; 3 Department of Chemical Engineering, Graduate School of Science and Engineering, Kagoshima University, 1-21-40 Korimoto, Kagoshima 890-0065, Japan; takei@cen.kagoshima-u.ac.jp; 4 Frontier Research Institute for Interdisciplinary Science, Tohoku University, 6-3 Aramaki aza Aoba-ku, Sendai, Miyagi 980-8578, Japan; gen@aerogel.jp

**Keywords:** composite liquid marble, core-shell bead, polymerase chain reaction (PCR)

## Abstract

Over the last three decades, the protocols and procedures of the DNA amplification technique, polymerase chain reaction (PCR), have been optimized and well developed. However, there have been no significant innovations in processes for sample dispersion for PCR that have reduced the amount of single-use or unrecyclable plastic waste produced. To address the issue of plastic waste, this paper reports the synthesis and successful use of a core-shell bead microreactor using photopolymerization of a composite liquid marble as a dispersion process. This platform uses the core-shell bead as a simple and effective sample dispersion medium that significantly reduces plastic waste generated compared to conventional PCR processes. Other improvements over conventional PCR processes of the novel dispersion platform include increasing the throughput capability, enhancing the performance and portability of the thermal cycler, and allowing for the contamination-free storage of samples after thermal cycling.

## 1. Introduction

Since the invention of polymerase chain reaction (PCR) in 1985 [1] this DNA amplification technique has gained extensive adaptation in various fields including biotechnology, cell biology, genetic engineering, forensic science, medical science, and drug discovery [2,3,4,5,6,7,8]. Various advancements and optimizations of PCR techniques such as quantitative reverse transcription PCR (qRT-PCR) and digital PCR have enabled scientific advancements in fundamental and applied molecular biology. Moreover, advances in microfluidics and optics have greatly improved the efficiency and detection limit of PCR techniques. However, very few updates have been reported in sample dispersion platforms for PCR. This lack of innovation means that sample dispersion platforms are limited to either conventional PCR tubes or conventional microfluidic chips based on polymers such as polydimethylsiloxane (PDMS) and polymethyl methacrylate (PMMA).

Urbina et al. estimated that bio labs around the world may have produced around 5.5 million tons of plastic waste in 2014 [9]. In light of the world side environmental challenges, this alarming figure motivates the development of alternative technologies to address the problem of plastic waste. Technological innovations should either eliminate or significantly reduce single-use plastic laboratory consumables. Biochemical and diagnostic assays such as PCR contribute to a significant share to the non-reusable contaminated plastic wastes. A single 0.2 mL PCR tube weighs approximately 0.14 g and a 100 µl pipette tip weighs approximately 0.3 g. Millions of conventional PCR tests are carried out in laboratories around the world annually, contributing towards the plastic wastes.

Conventional microfluidic chips for PCR poses the same problem of plastic waste. Lack of reusability of conventional microfluidic chips is the main disadvantage. Moreover, the design and development of conventional microfluidic devices are complex. Bulky external support systems such as pumps and tubing are required to deliver the samples into the microfluidic device, making many of them not suitable for practical field applications [10]. In this context, a suitable sample dispersion platform that has comparable performance to conventional PCR tubes and microfluidic devices but eliminating other disadvantages is of great interest.

Liquid marble, a liquid droplet coated with hydrophobic/oleophobic powder [11], is an ideal candidate for this purpose. The powder coating on the surface of the droplet isolates it from the surrounding, eliminating the possibility of contamination. Conventional liquid marbles possess reasonably good mechanical strength [12,13,14,15]. Moreover, the generation [16,17] and manipulation [18,19,20,21,22,23,24,25,26] of liquid marbles have been relatively well developed for other applications. In fact, significant advances in cell biology, bio-engineering, and bio-medicine have been made using liquid marbles as bio-reactors [27,28,29,30,31]. However, to our best knowledge, no significant advances in using liquid marbles for DNA amplification have been reported in the literature. The major issue of using a conventional liquid marble for polymerase chain reaction is the evaporation of the PCR mixture at elevated temperatures. We have previously developed a simple method to reduce evaporation of the PCR mixture in liquid marbles and successfully conducted PCR in a liquid marble coated with polytetrafluoroethylene (PTFE) powder [32]. However, the sample volume used in the experiment was relatively large (50 µl) and the PCR mixture could only last for 9 to 10 thermal cycles due to evaporation of PTFE-coated liquid marbles [33].

In this paper, we make use of core-shell beads synthesized from a composite liquid marble as a microreactor for carrying out PCR. The composite liquid marble consists of two immiscible liquid droplets forming a concentric spherical geometry and a coating of hydrophobic/oleophobic powder. Composite liquid marbles possess all the advantages of a conventional liquid marble, but also have to increase protection from external contamination of the core droplet by the shell liquid as well as the powder coating. Moreover, the shell liquid can transform through polymerization into a solid, converting the liquid marble into a core-shell bead. This versatility allows for convenient manipulation and storage of the liquid sample as a solid particle. We hypothesise that the shell liquid in its polymerised hardened state may also prevent the evaporation of interior liquid droplet at elevated temperatures, as the exterior solid polymer acts as a closed chamber. Here, we used the PCR mixture as the core liquid and a photopolymer as the shell liquid for manufacturing the composite liquid marble. The close packing of the exterior photopolymer shell liquid on the interior PCR mixture will considerably reduce the amount of plastic waste compared to conventional PCR approaches.

## 2. Materials and Methods 

### 2.1. Preparation of Photopolymer Liquid 

The photopolymer liquid is prepared by dissolving 0.05 g of camphorquinone and 0.06 g of ethyl-4-(dimethylamino) benzoate in 10 g of Trimethylolpropane trimethacrylate (TRIM) using a magnetic stirrer at 600 rpm for 2 min. The photopolymer liquid was stored in an opaque container for subsequent use. Trimethylolpropane trimethacrylate [34] is a crosslinking monomer which finds its applications in the field of dentistry and for the development of bone cement [35,36]. There is no cytotoxicity reported for the compound to the best of our knowledge. 

### 2.2. Synthesis of Composite Liquid Marbles and Core-Shell Beads 

**Figure 1:**
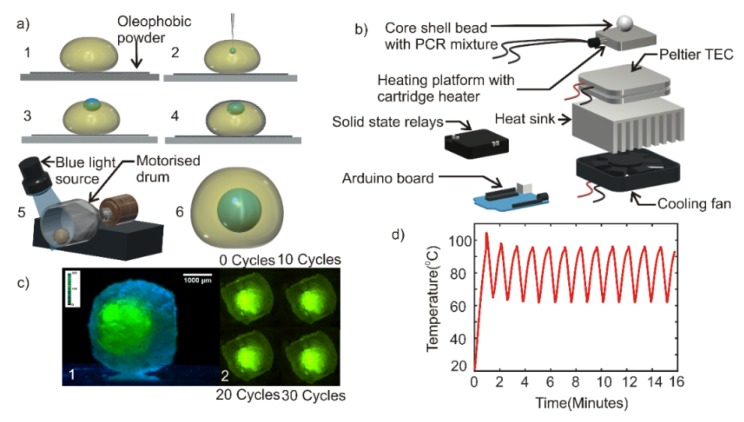
(**a**) (1) Deposition of photopolymer droplet on super amphiphobic powder bed. (2) Inserting polymerase chain reaction (PCR) mixture into the predeposited drop. (3) Inner droplet in a partially immersed state. (4) Amphiphobic powder-coated composite liquid marble. (5) Photopolymerization of composite liquid marble rotating at 140 rpm in a motorized drum. (6) Core-shell bead embedded with PCR mixture droplet. (**b**) Exploded view of the custom-built thermal cycler. (**c**) (1) Photograph of core-shell bead generated. (2) Photographs of the core-shell bead after at various cycles of thermal cycling. (**d**) Thermal cycles of the custom-built thermal cycler.

A super amphiphobic silicon monolith called “marshmallow-like gel” (MG) [37] was powdered using a mortar and collected in a plastic weighing container (35 mm × 35 mm × 10 mm), forming a super amphiphobic powder bed. A volume of 20 µl of the photopolymer prepared previously was deposited on to the powder bed using a micropipette as shown in Figure 1a, step 1. A volume of 2 µl of the PCR mixture was subsequently injected into the photopolymer (Figure 1a, step 2). The PCR mixture droplet was observed to be partially immersed in the polymer as shown in Figure 1a, step 3. Photopolymerization of the shell liquid at this stage would cause a part of the core droplet to be exposed to the ambient atmosphere, causing evaporation of the PCR mixture during the thermal cycling process. The composite liquid droplet obtained after step 3 was gently rolled on the powder bed to coat the powder on the liquid droplet, forming the composite liquid marble (Figure 1a, step 4). We observed that the powder coating on the liquid droplet had pushed the PCR mixture droplet into the shell polymer liquid due to the highly “phobic” nature of the PCR mixture to the powder as compared to the polymer liquid. 

Photopolymerization of the shell liquid at this stage would result in a core-shell bead with the PCR mixture encapsulated by the hardened polymer shell. However, the thickness of the hardened coating over the PCR mixture was relatively thin and could break due to thermal stress during the thermal cycling process. Hence, the composite liquid marble obtained after step 4 was transferred to a motorized cylindrical drum. The drum was rotated at 140 rpm. The rotating composite liquid marble inside the drum was illuminated with blue light and kept at a distance of 5 cm above the liquid marble for photopolymerization (Figure 1a, step 5). Takei et al. reported that the rotary motion of composite liquid marble would push the inner liquid droplet towards the center of the outer droplet [38]. The process of rotation and simultaneous photopolymerization was carried out for 5 min. A hardened core-shell bead with powder coating was obtained after 5 min. The powder on the surface of the bead was washed using de-ionized (DI) water to obtain a transparent core-shell bead (Figure 1a, step 6). Photographs of each of these steps and the core-shell bead generating set up is provided in the supplementary material (Figure S1a,b).

### 2.3. Design and Fabrication of Thermal Cycler

As a commercial thermal cycler for liquid marbles or core-shell beads does not exist, we developed a custom-built thermal cycler for the PCR experiment. A 20 mm × 20 mm × 15 mm aluminum block with embedded cartridge heater (5-mm diameter and 15-mm length, Core electronics) served as the heating platform. The aluminum heater block was attached to a 40 mm × 40 mm × 3.5 mm Peltier thermoelectric cooler (TEC-12706. AUS Electronics) using a heat conductive adhesive (Stars -922 heat sink plaster). The entire assembly was subsequently mounted on an aluminum heat sink (85.6 mm × 68.3 mm × 41.5 mm) of a computer CPU cooler fan (12 V, 3300 rpm, 70 mm × 70 mm × 25 mm) using heat conductive adhesive. An Arduino Mega microcontroller board was programmed to run a proportional-integral-derivative (PID) algorithm to control the temperature setpoints of the thermal cycle. Figure 1b shows the exploded view of the thermal cycler assembly. Photograph of the custom-built thermal cycler is provided in the supplementary material (Figure S1c).

A dummy experiment was carried out to test the characteristics of the thermal cycler and the performance of the core-shell bead. Core-shell bead containing a volume of 2 µl deionized water added with green fluorescent dye was prepared as per the methods explained in Section 2.2. The side view image of the core-shell bead was taken with a complementary metal oxide semiconductor Sensor (CMOS) camera (Edmund Optic EO-5012C) attached to a 1× telecentric lens (Edmund Optics), Figure 1c (1). The core-shell bead was placed on the heater platform of the thermal cycler. A thin layer of heat sink paste was affixed between the heater platform and the core-shell bead to ensure efficient heat transfer. The thermal cycler was operated for 30 cycles each cycle having the following conditions: 95 °C for 15 s and 60 °C for 45 s. The top-view image of the core-shell bead was taken with a CMOS camera (Edmund Optic EO-5012C) attached to a 0.5× telecentric lens (Edmund Optics), Figure 1c (2). The recorded image shows that the droplet inside the hardened bead was stable over the thermal cycles. The experiment was repeated in 3 core-shell beads to ensure repeatability.

The custom-built thermal cycler demonstrated a ramping rate of 0.68 K/s during heating and 1 K/s during cooling. A maximum overshoot of 8 K was shown in the first thermal cycle and stabilized during the subsequent thermal cycles. The steady-state temperature of the thermal cycle was observed to be 95 ± 0.7 °C and 60 ± 1.5 °C. Figure 1d depicts the performance of the thermal cycler. 

### 2.4. Preparation and Optimization of the PCR Mixture

DNA was extracted from the fecal sample of a healthy individual using the QIAmp DNA stool mini kit (Qiagen). This template DNA was used to synthesize artificial copies using a conventional PCR machine. Reverse primer sequence: 5ʹ CGTTACCCCGCCTACTATCTAATG-3ʹ and forward primer sequence: 5ʹ -TGAGTTCACATGTCCGCATGA-3ʹ were used for the detection of human-specific fecal DNA marker (BacHuman). The reaction was performed using GoTaq green master mix (Promega) under the following conditions: initial annealing at 50 °C for 2 min, 95 °C for 10 min, followed by 39 cycles at 95 °C for 15 s and 60 °C for 1 min. The artificial template DNAs were serially diluted to 100 ng/µl, 150 ng/µl and 200 ng/µl concentrations for the subsequent experiments. Samples were prepared in duplicate. One set of samples would be used to carry out core-shell based PCR while the other set would be used for qRT-PCR control experiments in a conventional commercial qRT-PCR machine.

### 2.5. Design and Fabrication of Fluorescent Detection System

The excitation and emission wavelengths of the PCR mixture used in the experiments are 450–490 nm (blue) and 520–560 nm (green) respectively. Intensity of green fluorescence emitted from the sample is proportional to the amplification efficiency of PCR. An LED lighting system was custom-built by arranging 30 blue LEDs (1500mCd, Jaycar, Brisbane, Australia) in concentric circles. This lighting source was used as the source of illumination in the proposed experiment. A CMOS camera (Edmund Optic EO-5012C) attached with a 0.5× telecentric lens (Edmund Optics-63074) mounted vertically was used to capture the green fluorescent light emitted from the core-shell bead. The green light emitted by the PCR mixture in the core-shell bead was optically filtered using a green optical filter (520–560 nm) for better signal to noise ratio.

## 3. Experimental 

**Figure 2:**
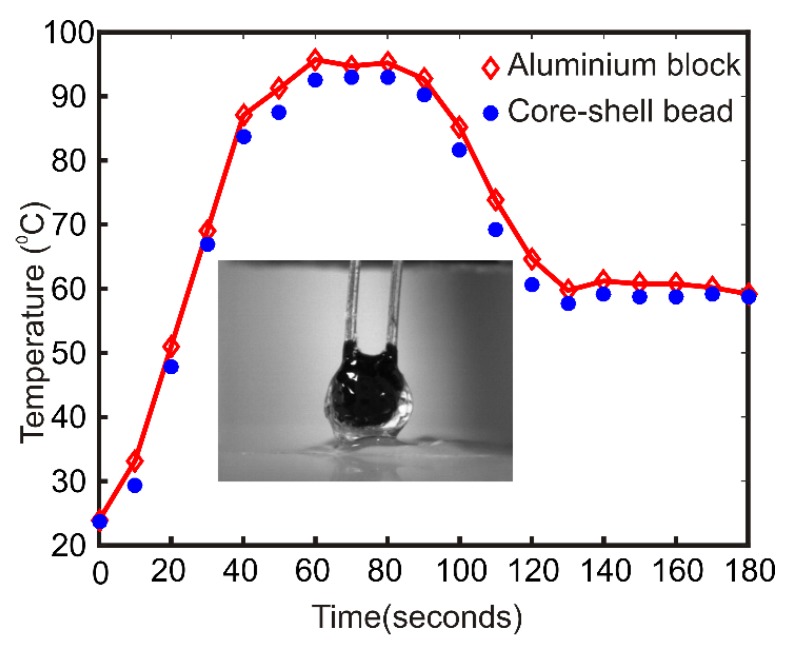
Comparison of heater block temperature with the polymer bead temperature. The inset picture shows the polymer bead with the thermistor.

The heat transfer characteristics of the core-shell bead is important for the efficient PCR inside the bead. A dummy experiment was carried out to test the heat transfer characteristics of the core-shell bead. A volume of 50 µl photopolymer liquid is deposited on the powder bed. A calibrated negative temperature coefficient (NTC) thermistor (Build Circuit, Australia) was vertically inserted into the photopolymer liquid droplet using a precision positioning stage. The photopolymer droplet was kept under the blue light source for 5 min for hardening. After a few minutes, a hardened bead embedded with the thermistor was obtained. The hardened bead along with the thermistor was placed on the aluminum heater block. The Arduino microcontroller of the thermal cycler was programmed to heat the aluminum block to 95 °C for 30 s and 60 °C thereafter forever. One heating and cooling cycle are observed. Figure 2 compares the temperature difference between the aluminum block and the polymerized bead during the thermal cycle. The inset shows the polymer bead with the NTC. The average temperature difference between the block and the bead was observed to be 2.12 K for the upper temperature and 1.31 K for the lower temperature.

**Figure 3:**
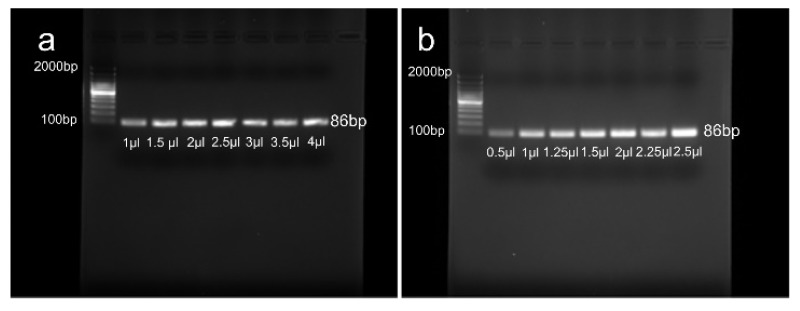
Gel electrophoresis results of (**a**) DNA optimization and (**b**) Primer optimization.

DNA and primer optimization were carried out to determine the right amount of PCR reagents. The reaction mixture (20 μL) for DNA optimization contained 10 µl GoTaq Green master mix, 1 μL (10 µM) of each forward and reverse primer, and a range of the template DNA including 1, 1.5, 2, 2.5, 3, 3.5, and 4 µl (100 ng/µl). The reaction mixture (20 μL) for primer optimization consisted of 10 µl GoTaq Green master mix, 2.5 μL (100 ng/µl) of template DNA, and a range of each forward and reverse primer (0.5, 1, 1.25, 1.5, 2, 2.25, 2.5 µl). The results demonstrate that the amount of 2.5 µl for DNA and primers is the optimum amount for the PCR in our experiment. Figure 3 shows the gel electrophoresis results of DNA and primer optimization.

**Figure 4:**
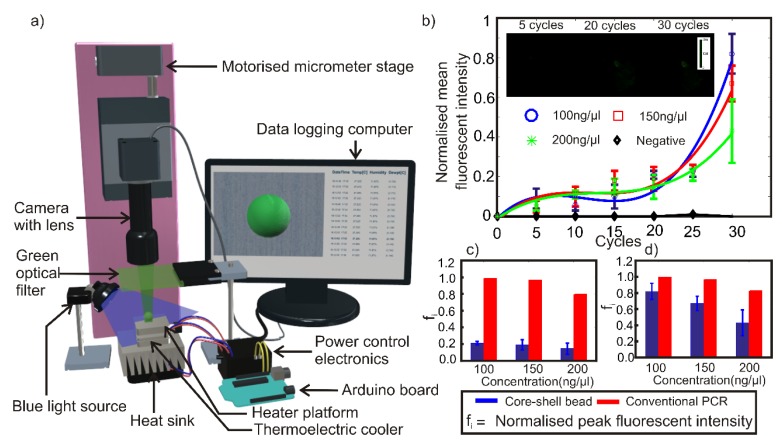
(**a**) Schematic of the experimental setup. (**b**) Variation of normalized mean fluorescent intensity of the PCR mixture in the core-shell bead with respect to thermal cycles. Sample micrographs of the core-shell bead are given as inset. Comparison of normalized peak fluorescent intensity of core-shell bead PCR with conventional quantitative reverse transcription PCR (qRT-PCR) after (**c**) 20 cycles. (**d**) 30 cycles.

A core-shell bead was prepared by using 2-µl PCR mixture as the core liquid and 20-µl polymer liquid as the shell liquid as described in Section 2.2. The core-shell bead containing the PCR mixture was kept on the heating platform of the thermal cycler. A thin layer of heat conductive paste was applied between the heating platform and the core-shell bead to ensure efficient heat transfer. The thermal cycler was subsequently turned on to run 95 °C for 15 s as one-time initiation and 30 thermal cycles having the following cycling conditions: 95 °C for 15 s and 60 °C for 45 s. The experiment was conducted in a dark laboratory environment at 23 °C and 50% relative humidity. Blue light from an LED light source was aimed at the composite bead, the light emitted from the bead was filtered using a green optical filter and video captured using a vertically mounted CMOS camera (Edmund Optic EO-5012C) attached with a 0.5x telecentric lens (Edmund Optics-63074). The blue light source was manually activated to illuminate the bead in intervals of 5 cycles. Switching off the excitation light reduces the possibility of photobleaching of fluorophores under continuous illumination. Experiments were repeated three times each for PCR mixture samples with 100 ng/µl, 150 ng/µl and 200 ng/µl DNA concentrations. Negative control for BacHuman was also prepared using S. aureus DNA. Experiments were repeated for negative control with similar thermal cycling conditions. Figure 4a illustrates schematically the experimental setup.

The average pixel density of the circular region containing the core-shell bead in the images was identified using ImageJ software. The values represent the numerical equivalent of the fluorescent intensities emitted by the bead at respective times. The numerical equivalent values of fluorescent intensities were offset corrected and normalized as:

(1) $$I_{sc}^{*}=\left(I_{sc}-I_{s0}\right)/I_{max}$$

Where $I_{sc}$ is the fluorescent intensity of a sample measured at a given cycle, $I_{s0}$ is the fluorescent intensity of that sample at the beginning of thermal cycling and $I_{max}$ is the maximum fluorescent intensity recorded among all the samples in the experiment. The standard error of the measurements in fluorescent intensity is also calculated using the equation:

(2) $$S.E=\sigma /\sqrt{n}$$

(3) $$\mathrm{and}\;\sigma =\sqrt{\frac{\sum^{\;}{\left(I_{sc}^{*}-I_{sc\_mean)}^{*}\right)}^{2}}{\left(n-1\right)}}$$

Where $I_{sc\_mean}^{*}$ is the average of normalised numerical equivalent values of fluorescent intensities measured for various samples of PCR mixture with a particular DNA concentration and n = 3 is the total number of samples tested. 

Weighing of the core-shell bead was performed subsequently. The weight of the 5 core shell beads containing the PCR mixture was measured individually using an electronic weighing balance (Entris 124I-1s, Sartorius Lab Instruments). Weights of five 0.2 mL conventional PCR tubes were also measured to compare the plastic waste reduction. Percentage reduction in non-re-usable contaminated plastic waste was calculated as

(4) $$\Delta w=\left(w_{t}-w_{b}\right)/w_{t}\times 100$$

Where $w_{t}$ is the weight of the conventional PCR tube (0.2 mL) and $w_{b}$ is the weight of the core-shell bead.

## 4. Results and discussion

The experimental results, Figure 4b–d show that there is a steady increase in fluorescent intensity as the thermal cycling process progresses and that the fluorescent results are comparable to, though not the same as, a conventional qRT-PCT method. Maximum fluorescent emission was detected from a sample with 100 ng/µl DNA concentration after 30 cycles. The peak fluorescent intensity of the samples was observed to decrease with increasing DNA concentration. The results confirm the amplification of DNA inside the core-shell bead. The negative samples did not show any significant fluorescence signal even after 30 thermal cycles. The fluorescent intensity values of samples were observed to be ~20% of the maximum fluorescent intensity throughout the first 20 thermal cycles, where the intensity then showed an exponential increase afterward up to a total of 30 cycles. Figure 4c,d depict the comparison of normalized peak fluorescent intensities of core-shell bead PCR and a conventional qRT-PCR. The result of fluorescent intensities from the conventional qRT-PCR shows that the optimum DNA concentration for optimum amplification efficiency is 100 ng/µl, matching that of core-shell bead PCR. The trend of the amplification efficiency with respect to the DNA concentration also matches with the observation derived from core-shell bead PCR within a reasonable band of error. It should be noted that the results depicted in Figure 4c,d are the comparisons of trends in DNA amplification efficiency with respect to the DNA concentrations. Fluorescent intensity values of core-shell bead PCR and conventional qRT-PCR cannot be compared in an absolute sense due to the following reasons. Firstly, a commercial machine uses a highly sensitive optical instrumentation system and algorithms to evaluate the fluorescence. Secondly, the method of core-shell bead synthesis is not completely optimized to ensure repeatability of the geometric location of the core PCR mixture inside the core-shell. Thirdly, minor variations in fluorescence will have a magnified effect on the off the shelf optical detection method used in this experiment. Fourthly, variations in size and shape of the core-shell bead, as well as the core PCR mixture, are also expected to make a difference in the fluorescent intensity. 

These results confirm that, despite this method being non-optimized and using off-the-shelf equipment, the core-shell dispersion method is a potential replacement for other dispersion methods used in PCR techniques. This method of manufacturing core-shell structures using composite liquid marble methods for sample dispersion and containment is able to protect the sample during thermal cycling and achieve fluorescence results comparable to the conventional method.

In addition, for the 10 experiments presented here, the conventional dispersion method produced approximately ~1.41 g of contaminated plastic waste, while the core-shell method produced ~0.21 g of contaminated plastic waste. The percentage reduction in contaminated plastic waste is ~85.1%. Sample pictures of the weight measurement are given in supplementary material (Figure S1d).

Although the increase in fluorescent intensity with respect to increasing thermal cycles is evidence of successful polymerase chain reaction, there are a number of interfering inputs that may affect the fluorescent intensity at any instant of time during the experiment. The volume of the PCR sample inside the core-shell bead, sensitivity and resolution of the camera-based detection system, transparency of the core-shell bead, geometrical location of the PCR sample inside the core-shell bead, uniformity in size, and shape of the core-shell bead are some of the interfering factors which can affect the fluorescent intensity measured during the experiment. It should be noted that the sample volume used in this experiment is only 2 µl. The sensitivity and resolution of the off the shelf camera-based fluorescent detection system used in this experiment is not good enough to distinguish the fine variations in fluorescence, which may be the reason for the “roughly equal” trend of fluorescent intensity curves until approximately 20 thermal cycles. 

This study has shown that this core-shell bead techniques can reduce plastic waste and improve the stability of samples in thermal cycling, however, further improvements and optimization of this method are still required. The variations in the geometrical position of the PCR sample inside the core-shell bead and uniformity in size and shape of the core-shell bead are two important factors affecting the quantification of fluorescent intensity. Although the method of core-shell bead production proposed in this paper successfully synthesized the core-shell beads, extensive characterization and optimization of the method, including the development of ideal compatible photopolymers with similar densities to the PCR mixture, has not been completed. The proposed core-shell bead preparation method in its optimized state should enable the synthesis of highly uniform core-shell beads which will reduce plastic waste produced during PCR techniques, as well as improving the storage ability of samples.

## 5. Conclusions

In this paper, a core-shell polymer bead was synthesized using composite liquid marble technology and subsequent photopolymerization aimed to reduce the contaminated plastic wastes and improve PCR methods of sample dispersion. A volume of 2 µl of PCR mixture with three different concentrations was successfully embedded in the core-shell beads. A customized thermal cycler was designed and fabricated to perform thermal cycling of the core-shell bead, and thermal cycling of the PCR mixture core-shell bead was performed and the first successful DNA amplification inside a core-shell bead using composite liquid marble technology was presented. It was observed that the core-shell bead prevents the evaporation of PCR liquid and allows an increased number of thermal cycles compared to previously presented PTFE liquid marbles. The mechanical handling of these hardened polymer core-shell beads is also easier compared to conventional liquid marbles. 

The method of generation of the core-shell bead using composite liquid marble technology is simpler compared to the conventional PDMS/PMMA based microfluidic chips. The proposed method was shown to reduce the plastic wastes generated in the conventional PCR sample dispersion methods by up to 85.1%. As the laboratory bench surface area required to conduct PCR of a 2-µl liquid marble is considerably less than the conventional method, this method can improve the throughput capability of the PCR substantially. The core-shell sample containment also offers contamination-free storage of the PCR sample after thermal cycling in an easy and space-efficient way. Although there is a considerable amount of plastic waste reduction in the proposed method, there is still a small amount of chemical waste generated in the proposed method. The amphiphobic marshmallow-like gel powder used to generate composite liquid marbles (prior to photopolymerization) is the chemical waste generated in the process. However, the amount of powder required to make one core shell bead is negligibly small as it only forms a thin layer of coating over the droplet. 

The core-shell beads based on liquid marble technology has potential application in both qRT-PCR and in digital PCR. However, the technology still has room to improve. Customized photopolymer with a similar density that of a PCR mixture should further reduce the time and complexity of processes involved in the synthesis of the beads. An optimized optical probing and fluorescence detection system may significantly enhance the detection limit of the proposed method.

Although our proof-of-concept-system generates a small amount of plastic waste and needs complex auxiliary setups, droplet-based PCR in conventional microfluidic chips promises very high throughput and this method is further developed [10]. The throughput capability of the core-shell bead PCR presented here is limited as the method of core-shell bead synthesis involves a significant amount of mechanical processes. The same could be improved by developing an automated core-shell bead generation system. Since it is a matter of photopolymerizing the exterior polymer liquid, conventional microfluidic droplet generation chips using PDMS or PMMA can be developed to make core-shell beads in applications where the issue of plastic waste and complexity due to pumps and tubing are insignificant compared to the required throughput capability. Conventional microfluidic droplet generation techniques are so advanced that it can generate droplets of picolitre (pL) volume [39]. These techniques could be explored to synthesize core-shell beads with much smaller volumes. 

The present paper reports the successful PCR of 2-µl mixture in 20-µl polymer core-shell bead. Variations in the volume ratios between interior and exterior liquids are expected to affect the performance and efficiency of the core-shell bead-making process as well as the efficiency of PCR. A significantly lower volume of PCR liquid compared to the exterior polymer liquid might affect the efficacy of thermal cycling. Sensitivity and resolution of the fluorescence detection system must be on the higher side to detect the fluorescence from a small volume of PCR liquid. On the other hand, a significantly larger volume of core liquid than the polymer liquid is expected to make it difficult to embed the liquid core inside the exterior shell. Although it is embedded, the exterior shell would become delicate and there is a higher possibility for the fragile shell to break at a higher temperature of thermal cycles. Characterization and optimization of the core-shell bead generation system for various core liquid to shell liquid volume ratios are necessary to establish a complete protocol for the proposed method. Characterization and optimization of the core-shell bead generation system are out of the scope of this paper; however, future studies should address these issues.

Sample retrieval after the thermal cycling is not possible in the proposed method as the shell material is hard in the polymerized state. Sample retrieval after the thermal cycling may be made possible by using a suitable reversible polymer as the shell material. Reversible polymers are those, which can be depolymerized back to original monomers under suitable conditions [40,41,42,43]. It should be noted that the proposed method was not optimized for the fluorescent intensity characterization of the PCR. Errors due to size and shape of the synthesized core-shell bead, errors due to the variation in geometrical location of the PCR sample inside the core-shell bead, inability of sample retrieval from the core-shell bead are the major hindrances towards the fluorescent intensity characterization and performance benchmarking of the proposed method. Sample retrieval from the micro reactor by using a reversible polymer as shell material along with the development of a highly repeatable and optimized core-shell bead generation system might significantly enhance the evaluation and calibration of the proposed core-shell bead PCR. Further research should be carried out in this direction.

## Supplementary Materials

The following are available online at https://www.mdpi.com/2072-666X/11/3/242/s1, Figure S1: (a) Photographs of various stages of core-shell bead production. (1) Deposition of photo polymer droplet on super amphiphobic powder bed. (2) Inserting fluorescent dye mixed water into the predeposited drop. (3) Inner droplet in partially immersed state. (4) Amphiphobic powder coated composite liquid marble. (5) Photo polymerization of composite liquid marble rotating at 140 rpm in a motorized drum. (6) Core-shell bead embedded with water droplet. (b) Photograph of the core-shell bead generating setup. (c) Photograph of the custom-built thermal cycler. (d) Photograph of weighing conventional PCR tube and core-shell bead.
