# Supplementary material for: Core-Shell Beads Made by Composite Liquid Marble Technology as A Versatile Microreactor for Polymerase Chain Reaction
Source: Micromachines (Basel). 2020 Feb 26;11(3):242. doi: 10.3390/mi11030242 (PMC7142426; doi:10.3390/mi11030242)
Supplement: Supplementary file 1 [file micromachines-11-00242-s001.pdf]

Supplementary Materials

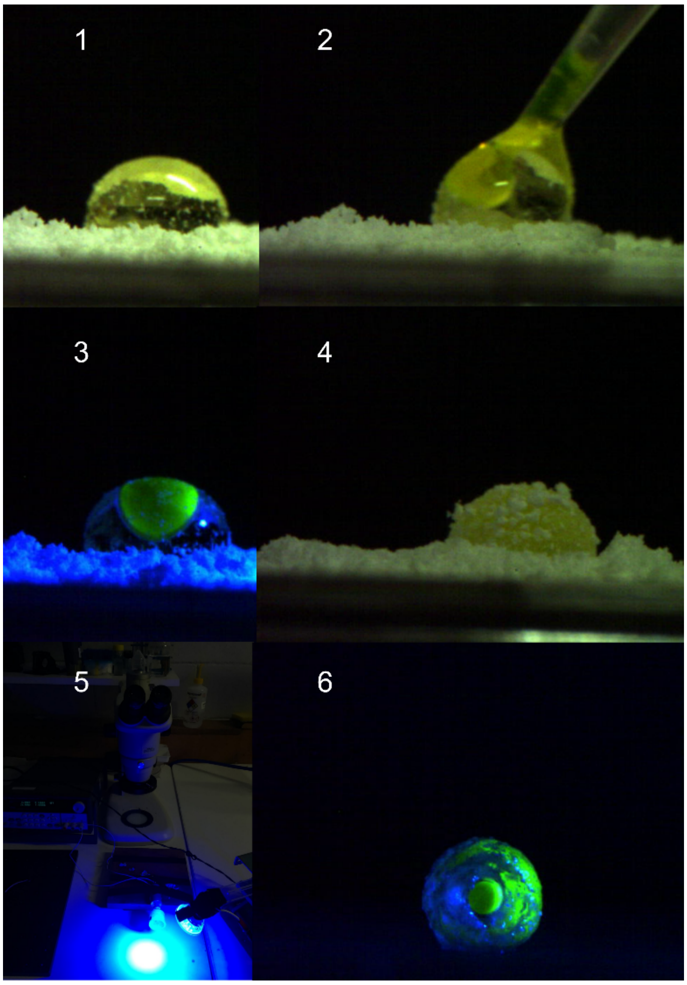

(**a**)

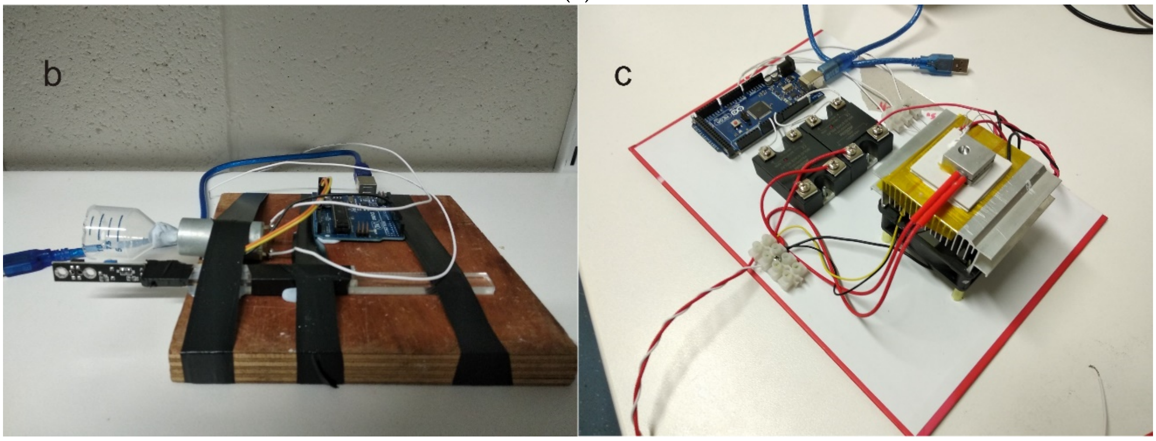

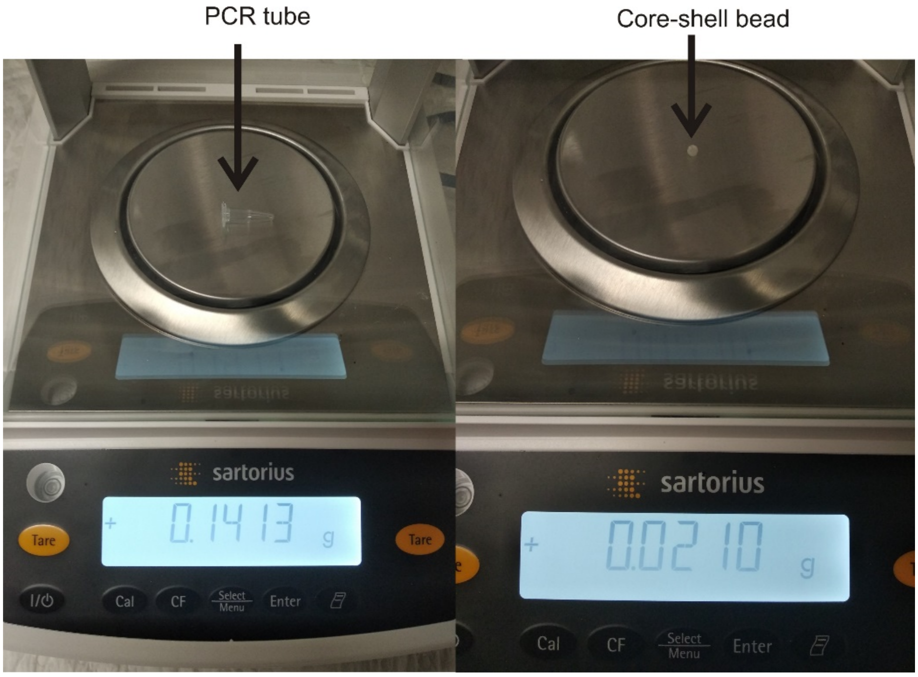

(**d**)

Figure S1. (**a**) Photographs of various stages of core-shell bead production. (1) Deposition of photo polymer droplet on super amphiphobic powder bed. (2) Inserting fluorescent dye mixed water into the predeposited drop. (3) Inner droplet in partially immersed state. (4) Amphiphobic powder coated composite liquid marble. (5) Photo polymerization of composite liquid marble rotating at 140 rpm in a motorized drum. (6) Core-shell bead embedded with water droplet. (**b**) Photograph of the core-shell bead generating setup. (**c**) Photograph of the custom-built thermal cycler. (**d**) Photograph of weighing conventional PCR tube and core-shell bead.
